# Augmin is a Ran-regulated spindle assembly factor

**DOI:** 10.1016/j.jbc.2023.104736

**Published:** 2023-04-21

**Authors:** Jodi Kraus, Sophie M. Travis, Matthew R. King, Sabine Petry

**Affiliations:** Department of Molecular Biology, Princeton University, Princeton, New Jersey, USA

**Keywords:** Ran pathway, branching microtubule nucleation, spindle assembly factor, importins, augmin, TPX2

## Abstract

Mitotic spindles are composed of microtubules (MTs) that must nucleate at the right place and time. Ran regulates this process by directly controlling the release of spindle assembly factors (SAFs) from nucleocytoplasmic shuttle proteins importin-αβ and subsequently forms a biochemical gradient of SAFs localized around chromosomes. The majority of spindle MTs are generated by branching MT nucleation, which has been shown to require an eight-subunit protein complex known as augmin. In *Xenopus laevis*, Ran can control branching through a canonical SAF, TPX2, which is nonessential in *Drosophila melanogaster* embryos and HeLa cells. Thus, how Ran regulates branching MT nucleation when TPX2 is not required remains unknown. Here, we use *in vitro* pulldowns and total internal reflection fluorescence microscopy to show that augmin is a Ran-regulated SAF. We demonstrate that augmin directly interacts with both importin-α and importin-β through two nuclear localization sequences on the Haus8 subunit, which overlap with the MT-binding site. Moreover, we show that Ran controls localization of augmin to MTs in both *Xenopus* egg extract and *in vitro*. Our results demonstrate that RanGTP directly regulates augmin, which establishes a new way by which Ran controls branching MT nucleation and spindle assembly both in the absence and presence of TPX2.

The mitotic spindle is a self-organized structure built from microtubules (MTs). Spindle MTs are made by several MT nucleation pathways that originate from various locations within the spindle (1, 2, 3). MT nucleation in the cell requires the universal MT template, the γ-tubulin ring complex (γ-TuRC) (4, 5). However, different nucleation pathways use unique factors to recruit γ-TuRC to the appropriate MT nucleation site, and this recruitment is strictly regulated in space and time to allow proper spindle assembly (6, 7). In centrosomal spindles, chromosomes themselves are a key source of MTs, and, in acentrosomal spindles, chromosomes are the major regulator of spindle assembly (8, 9, 10). Central to the chromosome’s ability to make spindle MTs is a biochemical gradient of RanGTP (11, 12).

Ran is a soluble small GTPase of the Ras family (13, 14). During interphase, Ran directs the movement of cargoes, *via* effector heterodimeric importins, by promoting cargo binding by importins in the cytoplasm and cargo release within the nucleus (8, 15, 16). Once the nuclear envelope breaks down, the Ran-importin system regulates the formation of the mitotic spindle by directly controlling the release of spindle assembly factors (SAFs) (8). In its active state, Ran is bound to GTP, which binds importin-β and causes importin-α to release nuclear localization signal (NLS)–containing cargoes, including SAFs (17, 18). The inactive GDP-bound Ran cannot bind the importin-αβ heterodimer and thus promotes substrate binding and, therefore, SAF sequestration (19, 20). Although Ran is distributed uniformly throughout the cell, RanGTP is concentrated at chromosomes because of chromosomal localization of its activating guanosine nucleotide exchange factor, RCC1 (11, 21). RanGTP diffuses away from chromosomes and encounters RanGTPase-activating proteins, allowing Ran to hydrolyze its bound GTP and become inactive (11). The RanGTP gradient generates a secondary mitotic SAF gradient, where free active SAFs concentrate near chromosomes, thereby exerting spatial control of spindle assembly (11, 22, 23).

One key SAF that connects the RanGTP gradient to MT nucleation is the targeting protein for Xklp2 (TPX2). TPX2 facilitates branching MT nucleation, whereby one MT is nucleated from a pre-existing one, enabling amplification of MTs throughout the spindle (24, 25). In fact, branching provides a majority of MTs in centrosomal spindles (26) and is the main source of MTs in acentrosomal spindles including *Xenopus laevis* (27, 28). Recent work showed that TPX2 forms a condensed phase that concentrates numerous branching factors at spindle MTs as well as unpolymerized tubulin that can be used to build new MTs (29). Binding of TPX2 to the importin-αβ heterodimer inhibits both MT binding and condensation, thus inactivating TPX2 (30). However, recent evidence suggests that regulation of TPX2 may not be sufficient to control branching. *In vitro* studies of both *Drosophila* and human branching MT nucleation showed that branching can proceed in the absence of TPX2 (31, 32), and in *Drosophila* cells, TPX2 is not essential (33). Thus, this begs the question of whether a second branching factor might be a Ran-regulated SAF.

The hetero-octameric augmin complex was first described in *Drosophila*, where subunits were shown to be required for robust spindle assembly (34). Knockdown or depletion of augmin *in vivo* leads to dramatic reduction in spindle MT density, particularly in kinetochore fibers, and results in defects in spindle polarity and chromosome segregation both in *Drosophila* as well as in vertebrates and plants (26, 34, 35, 36). Later, characterization of augmin *in vitro* demonstrated that augmin binds to the universal MT nucleator, γ-TuRC, at one end of the complex known as tetramer III (T-III), whereas the other end of the complex, tetramer II (T-II), recognizes and binds MTs (37, 38) (Fig. 1*A*). Within T-II, there are two MT-binding sites (37, 39, 40). The primary MT-binding site was localized to the augmin subunit Haus8, within its intrinsically disordered N terminus (37, 40), whereas a second minor MT-binding site was very recently located within the Haus6 subunit (39, 41). Thus, augmin promotes branching MT nucleation by recruiting γ-TuRC to the side of the MT (31, 32, 38, 42).

**Figure 1:**
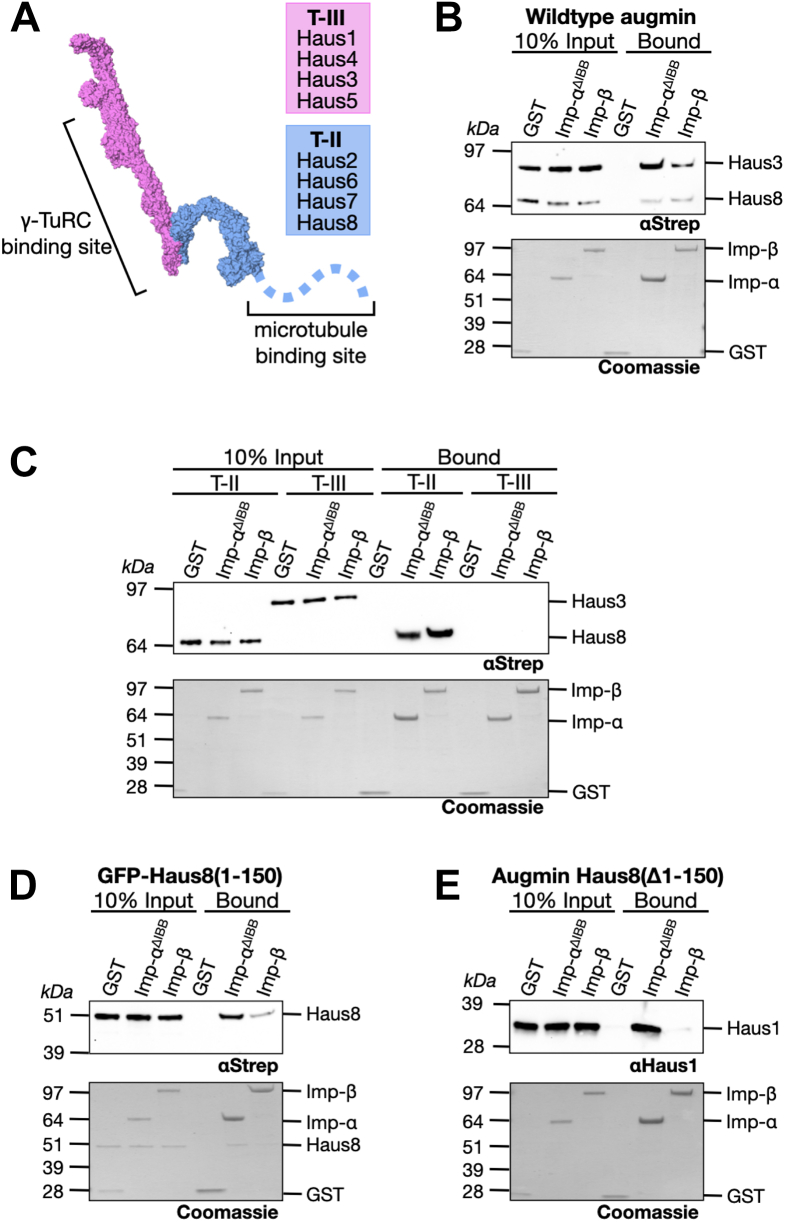
**Augmin binds to importins.***A*, structure of the augmin complex, broken into the γ-TuRC binding T-III (*pink*), comprised of subunits Haus1 and Haus3–5, and the MT-binding T-II (*blue*), comprised of subunits Haus2 and Haus6–8. The disordered N terminus of Haus8 (shown as a *dashed blue line*) contains the primary MT-binding site. Structure of *Xenopus* augmin was taken from Ref. (39). *B*, glutathione beads bound to either GST (control), GST-importin-α^ΔIBB^, or GST-importin-β were incubated with full-length augmin, then both the input and bound fraction were Western blotted for intact augmin complexes using an antibody against the Strep-tagged subunits Haus3 (T-III) and Haus8 (T-II). Below, GST and GST-importin loading was demonstrated by Coomassie stain. *C*, as in (*B*), importin-bound beads were incubated with augmin, either T-III or T-II, and binding of intact augmin subcomplex was detected *via* Western blot against the Strep-tagged subunits Haus3 and Haus8. *D*, Haus8^1–150^ (fused to an N-terminal Strep-tagged GFP) was incubated with importin-bound beads, and binding was detected *via* Western blot. *E*, augmin complex lacking the N-terminal 150 residues of Haus8 was incubated with importin-bound beads, and binding of intact augmin complex was detected *via* Western blot against augmin subunit Haus1. γ-TuRC, γ-tubulin ring complex; GST, glutathione-*S*-transferase; IBB, importin-β binding; MT, microtubule; T-III, tetramer III.

In this work, we demonstrate that the augmin complex, and more specifically, the N terminus of Haus8, has affinity for importin-αβ. This binding site, comprised of two conserved NLS sequences, overlaps with augmin’s MT-binding site in Haus8, allowing importin to modulate the ability of augmin to bind to MTs. We further demonstrate that Ran is capable of releasing augmin from sequestration by importins. Thus, we identify augmin as a second point of Ran regulation within the branching MT nucleation pathway and, because TPX2 is dispensable in many systems, propose that augmin may be the primary conserved point of Ran regulation in branching MT nucleation.

## Results

### Augmin binds to importins *via* T-II and the N terminus of Haus8

To determine whether augmin is a SAF, we first asked whether augmin is capable of binding importin-α and importin-β, a requirement of Ran regulation. Purified glutathione-*S*-transferase (GST)-tagged importins, as well as GST alone as a negative control, were immobilized on glutathione agarose resin and probed with recombinant *X. laevis* augmin complex. Because importin-α is autoinhibited by its NLS-like importin-β binding (IBB) sequence when not bound to importin-β (18), we used constitutively active importin-α lacking the IBB (residues 1–90), which we refer to as importin-α^ΔIBB^. We found that the augmin complex bound to both importin-α^ΔIBB^ and importin-β, with an apparent preference for importin-α^ΔIBB^ (Fig. 1*B*).

Because the augmin complex is large (∼450 kDa) and distinct functional roles are played by different subcomplexes (37, 38), we next asked where on the augmin complex the importin-binding site or sites were located. We started by purifying the two separate soluble augmin subcomplexes, known as T-II and T-III (Fig. 1*A*) (38). We found that only T-II had affinity for the two importins (Fig. 1*C*), binding both importin-α^ΔIBB^ and importin-β with approximately equal strength. In contrast, T-III displayed no appreciable binding to either importin (Fig. 1*C*). Next, we analyzed the sequences of the four T-II subunits (Haus2, Haus6, Haus7, and Haus8) for potential importin-binding sites (43). We found that the N terminus of Haus8 (residues 1–150) has many of the unique hallmarks of importin-binding regions, including an abundance of basic amino acids and, as a hallmark of disordered protein regions, a high percentage of serines and glycines (Fig. S1). Fortuitously, as opposed to many other subunits of augmin, the N terminus of Haus8 can be purified separately from the remainder of the augmin complex (40), and we found that this region does in fact bind both importin-α^ΔIBB^ and importin-β, again displaying a preference for importin-α^ΔIBB^ (Fig. 1*D*).

After showing that Haus8^1—150^ was sufficient to bind to importins, we determined whether this was the sole importin-binding site on augmin by expressing and purifying *Xenopus* augmin lacking Haus8^1—150^ and conducting the binding assay. Interestingly, we found that the truncated complex was still able to bind to importin-α^ΔIBB^, although importin-β binding was abrogated (Fig. 1*E*). Based on our binding experiments with the two tetrameric subcomplexes, we suspect that this second binding site must be located somewhere within T-II, since importins do not bind T-III. However, we could not determine the location of this second importin-α binding region because of the lack of obvious predicted NLS sequences and because the entwined structure of the remaining T-II subunits prevents any other augmin fragments from folding correctly and thus retaining function (39, 41, 44).

### Haus8 contains two predicted NLS sequences that mediate importin-α binding

**Figure 2:**
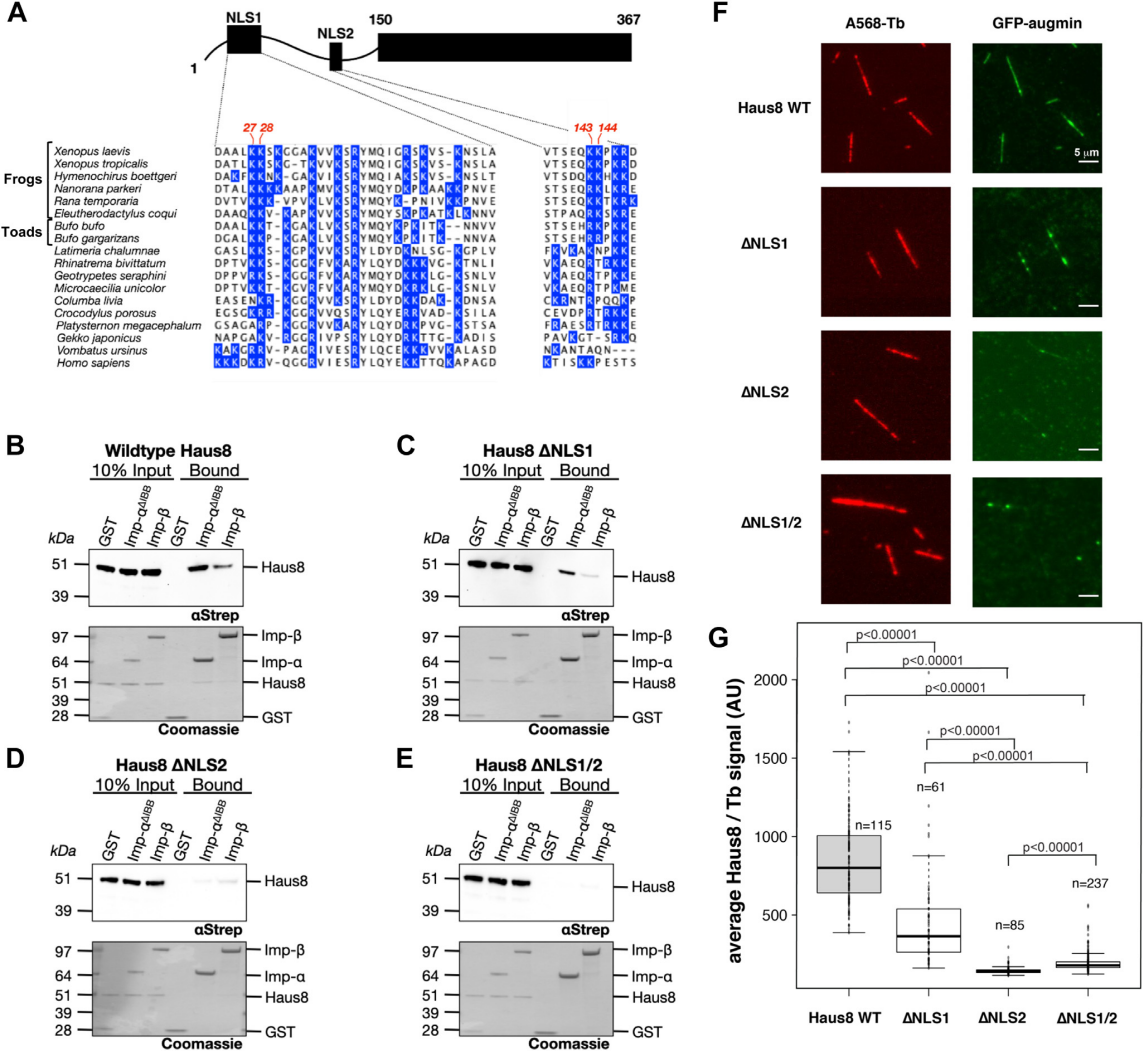
**Haus8 of augmin binds to importins and MTs through two NLS sites.***A*, the augmin subunit Haus8 is predicted to contain two NLS sequences within its disordered N terminus (43). The domain architecture of Haus8 is cartooned at *top*, and the sequence of each predicted NLS in *Xenopus laevis* is shown at the *bottom*. *X. laevis* Haus8 is shown aligned to other vertebrate orthologs below, and all basic residues (arginine abbreviated as R and lysine as K) are highlighted in *blue*. Indicated in *red* at the *top* of the sequence are the pairs of basic residues mutated to alanine to generate the Haus8 mutants ΔNLS1 (K27A/K28A) and ΔNLS2 (K143A/K144A). *B*–*E*, pulldowns of Strep-GFP-Haus8^1–150^, either wildtype or containing the indicated NLS mutants, were conducted as for Figure 1*D*. *F*, selected TIRF images of *in vitro* binding of GST-GFP-Haus8^1–150^ to stabilized MT seeds. Haus8 constructs with mutated residues in NLS1 and/or NLS2 result in a reduction in binding, as quantified in *G*. Images belonging to the same experiment were contrast matched. To compare augmin fluorescence intensity across experiments, the intensity was normalized with respect to the tubulin signal. *G*, boxplot of average GFP-Haus8 signal relative to the average tubulin signal, where each marker represents a single MT from the experiment shown in *F*. The total number of MTs (n) was collected from two replicates. *Center lines* show the medians; *box limits* indicate the 25th and 75th percentiles as determined by R software; *whiskers* extend 1.5 times the interquartile range from the 25th and 75th percentiles. *p* Values were calculated from independent *t* tests. GST, glutathione-*S*-transferase; MT, microtubule; NLS, nuclear localization signal; TIRF, total internal reflection fluorescence.

Having identified an importin-binding region within Haus8, we sought to characterize exactly where those sites are located. Using an NLS prediction algorithm (43), we found two putative NLS sites in the N terminus of Haus8, one comprising residues 23 to 54 and the other residues 138 to 148 (Fig. 2*A*). The first putative NLS is bipartite and contains residues that are highly conserved among most vertebrates, whereas the second monopartite NLS is predicted only in frogs and toads (Fig. 2*A*).

To test whether one or both of these two putative NLS sites are required for importin binding, we mutated the NLSs by introducing double alanine mutations at either Lys-27 and Lys-28 or Lys-143 and Lys-144 (45, 46). We observed that mutation of either NLS led to a decrease in importin-α^ΔIBB^ binding relative to wildtype (Fig. 2*B*). Moreover, mutation of NLS1 (Fig. 2*C*) had a lesser impact on importin binding than mutation of NLS2 (Fig. 2*D*). This may result either from a difference in importin-α binding affinity between the two NLS sequences, or, alternatively, may be because mutation of two residues in a bipartite NLS is less deleterious than mutation of two residues in a shorter and monopartite NLS. Nonetheless, combining mutations to both NLS sites completely abrogated importin-α^ΔIBB^ binding (Fig. 2*E*). In contrast, mutation of either or both NLS sequences had little to no measurable effect on importin-β binding (Fig. 2, *B*–*E*). Thus, Haus8 contains two NLS regions recognized primarily by importin-α.

In the previously studied proteins, TPX2 and NuMA, the NLS sites overlap with the MT-binding domains (30, 47, 48, 49). To test whether this is the case for augmin as well, we performed *in vitro* MT-binding assays using biotinylated stabilized MT seeds attached *via* neutravidin to functionalized glass coverslips. GFP-labeled Haus8 NLS mutants were diluted to 100 nM, which mimics the endogenous concentration, and incubated with immobilized MT seeds. The degree of binding was assessed by total internal reflection fluorescence (TIRF) microscopy. We found that single NLS mutants decreased the binding to MT seeds, whereas the double mutant lacking both NLS sites inhibited binding altogether (Fig. 2, *F* and *G*). Thus, the MT-binding sites in Haus8 overlap with the NLS regions, which is a common feature of other MAPs including NuMA and TPX2 (47, 49).

### Importins prevent augmin from binding the MT lattice

**Figure 3:**
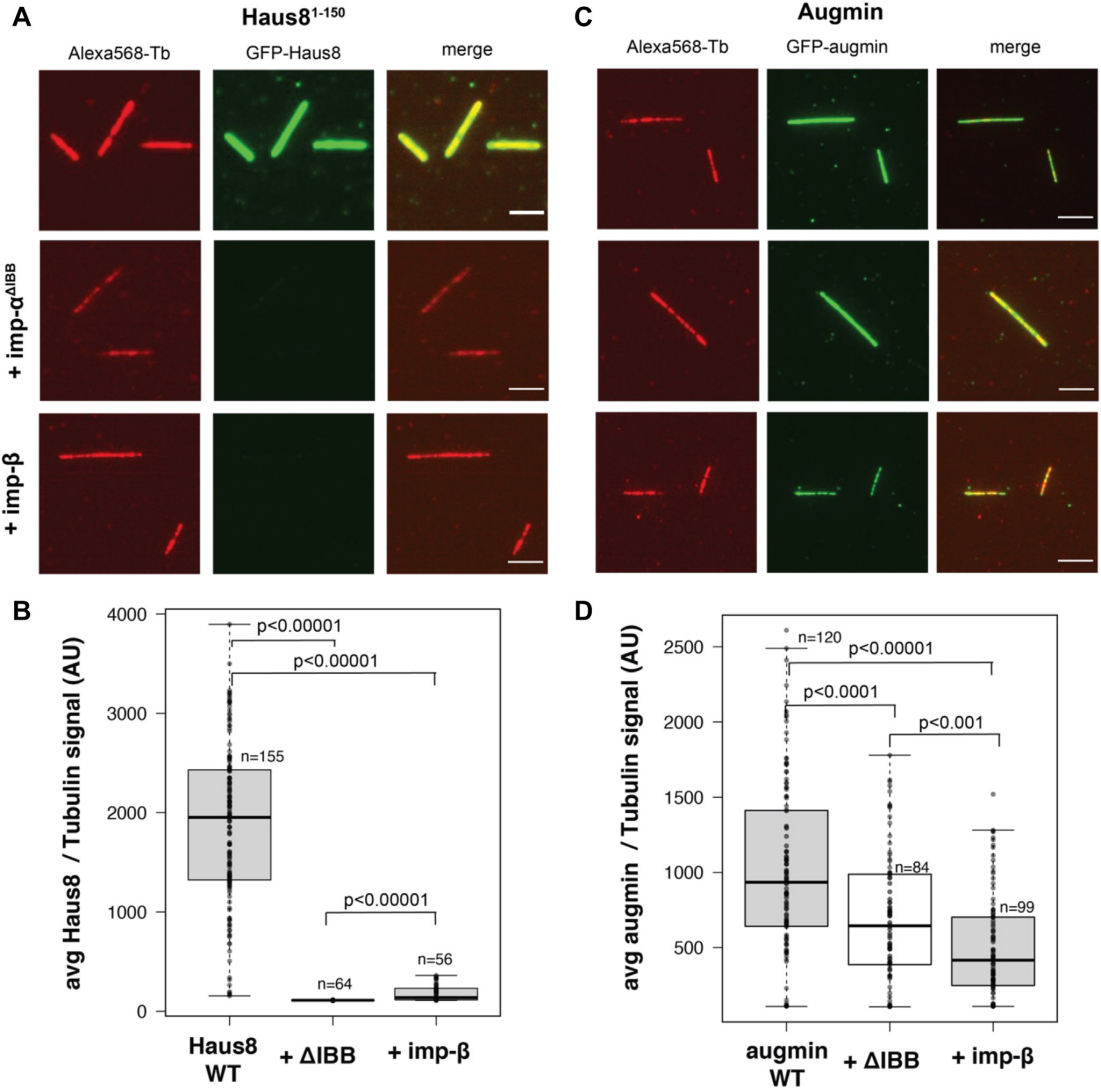
**Importins regulate augmin binding to MTs.***A*, WT GST-GFP-Haus8 localizes strongly to GMPCPP-stabilized MT seeds *in vitro* (*top row*), as visualized by TIRF microscopy. In the presence of importin-α^ΔIBB^ (*middle row*) or importin-β (*bottom row*), binding of Haus8 to MTs is diminished. This is quantified in *B*. *B*, boxplot of average GFP-Haus8 signal relative to the average tubulin signal, where each marker represents a single MT from the experiment shown in *F*. The total number of MTs (n) was collected from two replicates. The *boxes* extend from the 25th to 75th percentile, and the *upper* and *lower* bars represent the minimum and maximum, respectively. *p* Values were calculated from independent *t* tests. *C*, WT GFP-labeled augmin localized to GMPCPP-stabilized MT seeds *in vitro* (*top row*), as visualized by TIRF microscopy. In the presence of importin-α^ΔIBB^ (*middle row*) or importin-β (*bottom row*), binding of augmin to MTs is decreased but not eliminated. This is quantified in *D*. *D*, boxplot of average GFP-augmin signal relative to the average tubulin signal, where each marker represents a single MT from the experiment shown in *F*. The total number of MTs (n) was collected from two replicates using two independent augmin preparations. *Center lines* show the medians; *box limits* indicate the 25th and 75th percentiles as determined by R software; *whiskers* extend 1.5 times the interquartile range from the 25th and 75th percentiles. *p* Values were calculated from independent *t* tests. In *A* and *C*, images belonging to the same experiment were contrast matched. To compare Haus8 fluorescence intensity across experiments, the intensity was normalized with respect to the tubulin signal. Scale bars correspond to 5 μm. GST, glutathione-*S*-transferase; IBB, importin-β binding; MT, microtubule; TIRF, total internal reflection fluorescence.

A critical role of augmin is to bind to the side of the MT lattice to recruit γ-TuRC for branching MT nucleation. We hypothesized that this function may be Ran regulated and therefore asked whether importin binding to augmin modulated its interactions with MTs. To address this, we performed *in vitro* MT-binding assays with GFP-labeled Haus8. GFP-labeled Haus8 was incubated with a 10-fold molar excess of importins and then flowed onto the glass, followed by quantification of MT binding by TIRF microscopy. Interestingly, both importin-α and importin-β abrogated binding to MT seeds, indicating that importins indeed regulate augmin’s ability to bind to MTs (Fig. 3, *A* and *B*).

We next tested how importins affected MT binding in the context of the full eight-subunit augmin complex to see if this behavior was reproduced. Interestingly, addition of both importin-α and importin-β significantly decreased binding of the full-length augmin to the MTs but did not inhibit it altogether (Fig. 3, *C* and *D*). Therefore, this suggests that the secondary MT-binding site in the Haus6 subunit is insensitive to importin binding and still allows some localization to MTs. The functional consequences of the binding site in Haus6 are still under active investigation.

### Active Ran releases augmin from importins to promote MT binding *in vitro* and in *Xenopus* egg extract

**Figure 4:**
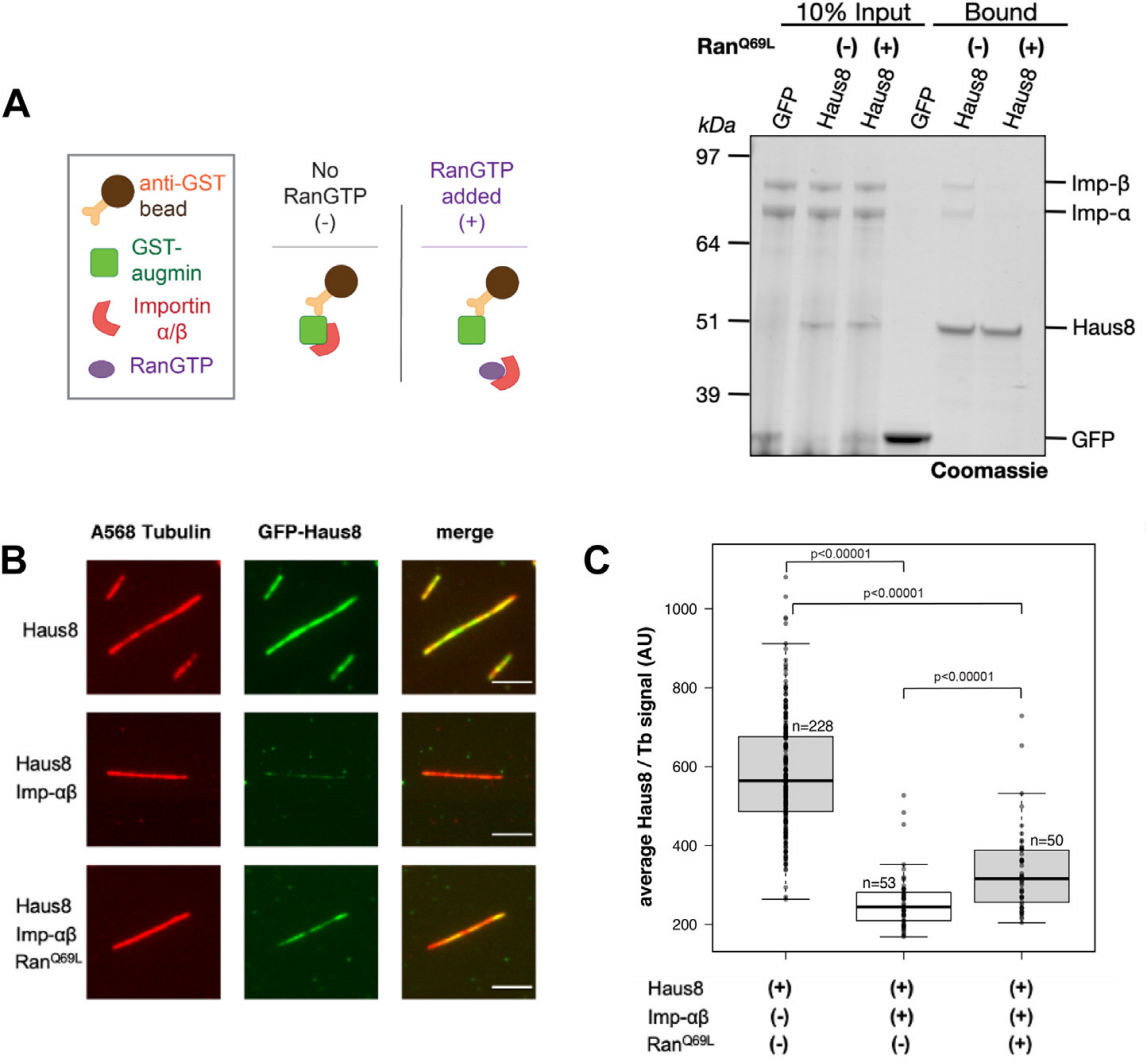
**RanGTP releases importin inhibition of MT binding.***A*, GFP or GFP-Haus8^1–150^ was bound to α-GFP magnetic resin and incubated with importin-αβ in the presence or the absence of a 10-fold excess of Ran^Q69L^. Both importin-αβ binding and augmin loading were assessed by Coomassie staining and the intensity of bands at the indicated sizes. *B*, *in vitro* localization of GST-GFP-Haus8^1–150^ binding to GMPCPP-stabilized MT seeds, as visualized by TIRF microscopy (*top row*). Addition of importin-αβ heterodimer inhibits binding of Haus8 to MTs (*middle row*), whereas addition of Ran^Q69L^ rescues MT binding of Haus8 (*bottom row*). Images belonging to the same experiment were contrast matched. To compare Haus8 fluorescence intensity across experiments, the intensity was normalized with respect to the tubulin signal. This is quantified in *C*. *C*, boxplot of average GFP-Haus8 signal relative to the average tubulin signal, where each marker represents a single MT from the experiment shown in *F*. n corresponds to the total number of MTs. *Center lines* show the medians; *box limits* indicate the 25th and 75th percentiles as determined by R software; *whiskers* extend 1.5 times the interquartile range from the 25th and 75th percentiles. *p* Values were calculated from independent *t* tests. Scale bars correspond to 5 μm. GST, glutathione-*S*-transferase; MT, microtubule; TIRF, total internal reflection fluorescence.

SAFs are not only characterized by importin inhibition but also by the ability of RanGTP to release this inhibition, thereby activating the SAF. To assess whether Ran regulates MT binding of augmin, we tested whether augmin binds the importin-αβ heterodimer, which, in contrast to monomeric importins, is the predominant form of importins taken in the cell. To test this, we pulled down importin-αβ heterodimer using beads loaded with GFP-Haus8^1–150^. We found that the N terminus of Haus8 bound strongly to a stoichiometric heterodimer of importin-α and importin-β relative to beads loaded only with GFP (Fig. 4*A*). When to the mixture was added a 10-fold excess of Ran^Q69L^, a mutant of Ran locked in a GTP-bound conformational state, importin-αβ was lost from the resin, implying that Ran^Q69L^ triggers the release of augmin from importin-αβ (Fig. 4*A*). Furthermore, importin-αβ inhibited binding of GFP-Haus8 to MT seeds *in vitro* under the same conditions, which was also rescued by the addition of active Ran^Q69L^ (Fig. 4, *B* and *C*).

**Figure 5:**
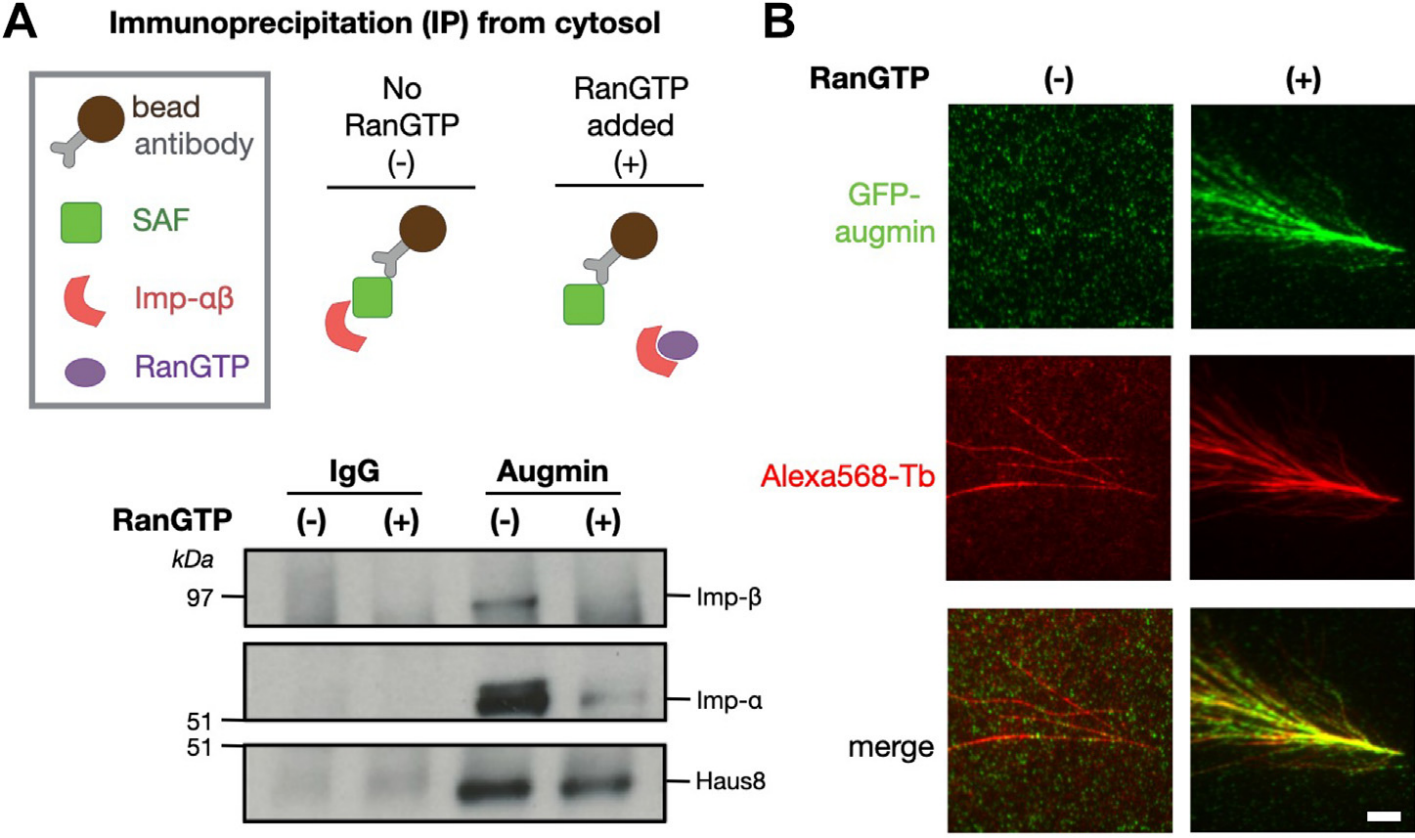
**Ran regulates augmin in *Xenopus* egg extract.***A* (*top*), schematic of immunoprecipitation (IP) strategy where antibodies were conjugated to magnetic beads (*top*). *Bottom*, Western blot of IPs for a control antibody (immunoglobulin [IgG]) and antiaugmin in the presence or the absence of Ran^Q69L^. *B*, TIRF images of augmin in *Xenopus* egg extract in the absence (*left column*) and presence (*right column*) of Ran^Q69L^. Branching MT nucleation reactions were carried out with Alexa568-labeled tubulin and fixed after 15 min. Endogenous augmin was detected *via* indirect immunofluorescence with custom primary antibodies against Haus8 and Alexa488-conjugated secondary antibodies. Scale bar corresponds to 5 μm. MT, microtubule; TIRF, total internal reflection fluorescence.

To determine whether our results *in vitro* were representative of Ran regulation of augmin in a physiological environment, we next assessed the role of Ran in regulating augmin in *X. laevis* egg extract. To test this, we first took advantage of an antibody against Haus1 that pulls down the augmin complex without disrupting endogenous binding interactions with proteins including γ-TuRC (38). Pulling down with this antibody, we found that substantially more importin-α and importin-β were bound to beads coated with α-Haus1 than nonspecific immunoglobulin G (IgG) (Fig. 5*A*), suggesting that augmin and importin-αβ interact in an endogenous setting. In addition, binding of endogenous importin-αβ was regulated by Ran, as incubation of the beads with extract spiked with Ran^Q69L^ substantially reduced the amount of importin pulled down (Fig. 5*A*). Finally, we generated branched MT structures in *X. laevis* egg extract and, using antibodies against Haus8 to visualize endogenous augmin, we found that augmin does not localize to MTs unless Ran^Q69L^ is present (Fig. 5*B*).

## Discussion

Here, we have shown that augmin is a *bona fide* SAF regulated by RanGTP. Augmin interacts with importin-α and importin-β and independently binds each importin through two NLS sites, which we identified. The NLS sites are located in the disordered N terminus of Haus8, which contains the primary MT-binding site, indicating that the NLS sites and MT-binding site(s) overlap. Moreover, importins inhibit binding of augmin to MTs, which can be reversed by addition of active RanGTP. Moreover, this regulatory system can be recapitulated both *in vitro* and in *Xenopus* egg extract.

While many studies have identified potential SAFs, none have previously recognized augmin as a candidate. High-throughput screens for importin clients in mammalian cells were limited to nuclear proteins (50); therefore, unsurprisingly, augmin was not identified, as during interphase augmin localizes to the centrosome and is excluded from the nucleus (36, 51). Other examples of SAFs that, during interphase, do not enter the nucleus include Kif2, which localizes to interphase MTs, and GM130, which localizes to the Golgi (8). Permeability of the nuclear pore complex to importin-bound cargos decreases linearly with cargo mass, with the most efficient transport of proteins under 40 kDa (52, 53). This would explain why proteins like TPX2 can easily shuttle in and out of the interphase nucleus but protein complexes like Kif2, GM130, and augmin cannot. Thus, many unidentified SAFs may exist, and their identification may further explain how spindle assembly is regulated.

While our identification of augmin as a target of Ran regulation is new and unexpected, a rich literature exists to support Ran regulation of branching MT nucleation, through TPX2 (30, 54, 55, 56). Why might Ran separately regulate two branching factors? As discussed previously, TPX2 is dispensable in certain systems, for example, spindle assembly in *Drosophila*, which might be taken to suggest that, in these systems, Ran regulates branching exclusively through augmin. However, *Drosophila* augmin lacks the NLS-containing disordered N terminus of its Haus8 subunit (known as Dgt4) and, in fact, previous work has suggested that, in *Drosophila*, augmin is not an importin client protein (57). Thus, in these systems, it is more likely either that branching MT nucleation is independent of Ran or, as has been shown in *Drosophila*, is less reliant on branching MT nucleation for spindle assembly compared with, for example, human cells.

Conversely, in *Xenopus* and other species, both TPX2 and augmin are required for branching, and thus their regulation by Ran would seem to be redundant. Yet, true redundancy is a core organizational principle of spindle assembly, because it is so important for the cell’s survival. Redundancy can be seen from the single protein level, in the multiple NLSs found in augmin and TPX2 (58), to the redundant functions of the various MT nucleation pathways. However, in addition, this dual control mechanism could be used to further sharpen the activity gradient of branching MT nucleation generated by RanGTP and therefore provide more finely tuned spatiotemporal control of the pathway generating the majority of spindle MTs (26).

In conclusion, our work, in identifying augmin as an Ran-regulated SAF, reveals another level of Ran control of branching MT nucleation, the major source of spindle MTs in vertebrate mitosis. However, it also raises larger questions in the regulation of spindle assembly as a whole, including how many unknown SAFs still are waiting to be identified and how simultaneous Ran regulation of multiple SAFs shapes the landscape of MT nucleation during mitosis to, finally, generate the dynamic and complex structure of the mitotic spindle.

## Experimental procedures

### Cloning

*X. laevis* Haus8 codon-optimized for *Escherichia coli* was synthesized by MacroLab and cloned into a pET3-based GFP-containing vector. N-terminal fragments were then subcloned *via* restriction enzyme cloning into modified pETDuet1 or pGEX4t1. NLS mutants were introduced through a modified QuickChange protocol (59). Recombinant bacmids were created by transformation into DH10Bac-competent cells (NEB), screening on XGAL plates, followed by bacmid isolation (60) and confirmation through diagnostic PCR.

### Protein expression and purification

N-terminal Haus8 fragments, tagged either with Strep-GFP or GST-GFP, were expressed in Rosetta 2 (DE3) pLysS-competent cells (NEB) grown in modified Terrific Broth at 27 °C for 7 h, following induction at midlog with 0.5 mM isopropyl β-d-thiogalactopyranoside. After harvesting, cells were resuspended in lysis buffer (50 mM Tris [pH 7.5], 750 mM NaCl, 5 mM EDTA, 1 mM DTT) supplemented with 10 μg/ml DNase I from bovine pancreas (Roche) and 1 mM phenylmethylsulfonyl fluoride. Cells were lysed using an Avestin Emulsiflex C5 and then clarified by ultracentrifugation at 105,000*g* at 4 °C for 30 min. Clarified Strep-GFP-Haus8 lysate was batch loaded onto 2 ml StrepTactin Superflow resin (IBA) per liter of cells, washed with 50 column volumes of lysis buffer, and eluted with five column volumes of lysis buffer supplemented with 2.5 mM desthiobiotin. GST-GFP-Haus8 constructs were purified similarly, except using glutathione Superflow agarose resin (Pierce) and eluting with lysis buffer supplemented with 10 mM reduced glutathione instead of desthiobiotin. After elution, samples were concentrated using a 30 kDa molecular weight cutoff spin concentrator (Amicon) to 1 ml and loaded onto a Superdex 200 Increase 10/300 column (Cytiva) pre-equilibrated with high-salt CSFxB (10 mM Hepes [pH 7.7], 500 mM KCl, 5 mM ethylene glycol-bis (β-aminoethyl ether)-N,N,N′,N′-tetraacetic acid, 1 mM MgCl_2_, and 1 mM DTT). Fractions were pooled and concentrated prior to flash freezing in liquid nitrogen and storage at −80 °C. Protein concentrations were determined by Bradford concentration assay using bovine serum albumin to generate a standard curve.

GST-tagged importins were previously described (29) and purified similarly, except substituting a lysis buffer with 200 mM NaCl and a gel filtration buffer with 100 mM KCl (low-salt CSFxB). Heterodimeric GST-importin-α bound to untagged importin-β was purified through separate expression and colysis of equal mass cell pellets of the two importins. Excess GST-importin-α was separated from stoichiometric heterodimer through gel filtration chromatography.

His-human Ran^Q69L^ was purified as previously described (61).

Heterologous expression of *X. laevis* augmin complex was performed in Sf9 cells as previously described (38). Full-length augmin was expressed either with an N-terminal HRV3C-cleavable ZZ-tag on Haus6 and a C-terminal GFP-His tag on Haus2 as previously described, or with two additional Strep-GFP tags on both Haus3 and Haus8. T-II was expressed with an N-terminal ZZ-tag on Haus6, a C-terminal GFP-His tag on Haus2, and an N-terminal Strep-GFP tag on Haus8. T-III was expressed with an N-terminal Strep-GFP tag on Haus3. All complexes were purified in augmin lysis buffer (50 mM Tris [pH 7.5], 200 mM NaCl, 5 mM EDTA, 2 μM β-mercaptoethanol, 10% [v/v] glycerol, and 0.05% [v/v] Tween-20) supplemented with 10 μg/ml DNase I and one cOmplete protease inhibitor cocktail tablet (Roche) per liter buffer. After lysis by Emulsiflex as aforementioned, lysates were clarified by ultracentrifugation at 250,000*g* at 4 °C for 30 min. Clarified full-length and T-II lysates were batch loaded onto 1 ml IgG Sepharose resin, washed with 50 column volumes of lysis buffer, and eluted overnight by GST-HRV3C protease cleavage. T-III was purified similarly, except substituting StrepTactin resin and desthiobiotin elution as aforementioned. Following elution, complexes were either concentrated using a 50 kDa molecular weight cutoff concentrator and further purified on a Superose 6 Increase 10/300 column (Cytiva) equilibrated in low-salt CSFxB or loaded directly onto Ni^2+^ agarose (Qiagen), eluted using 300 mM imidazole, then dialyzed overnight into low-salt CSFxB supplemented with 10% (w/v) d-sucrose.

### Pulldowns

For GST-importin-α^ΔIBB^ and GST-importin-β pulldowns, 30 μl of magnetic glutathione agarose (Pierce) was washed three times with 300 μl Tris-buffered saline (TBS) supplemented with 0.1% (v/v) Tween-20 (TBS-T), then equilibrated into 300 μl low-salt CSFxB supplemented with 0.1% (v/v) Tween-20 (CSFxB-T). About 30 μl of freshly made binding reaction, containing either 2 μM GST or GST-importin and either 2 μM GFP-Haus8 (1–150) or 200 nM augmin complex, were added to the beads, allowed to mix, and then incubated at 4 °C for 2 h. Supernatant was then removed, and beads were washed three times with 300 μl CSFxB-T. Finally, beads were resuspended in 30 μl CSFxB-T, mixed with 10 μl 4× SDS-PAGE loading dye, and heated at 95 °C to denature and elute any bound proteins. After SDS-PAGE, gels were either stained by Coomassie to detect total protein content or transferred to nitrocellulose *via* iBLOT (Invitrogen) and probed for augmin using either murine α-Strep (Qiagen) or a previously published rabbit α-*Xenopus* Haus1 (38).

For pulldowns with GST-importin-α bound to untagged importin-β, 30 μl of GFPTrap magnetic agarose (Chromotek) was substituted for aforementioned glutathione resin, and TBS supplemented with 0.1% (v/v) Tween-20 and CSFxB-T were supplemented with 10 mg/ml κ-casein; however, the remainder of the pulldown proceeded in the same manner.

For pulldowns of endogenous augmin, *Xenopus* egg extract was prepared as previously described (38, 61). Studies were approved by the Princeton University Institutional Animal Care and Use Committee. α-Haus8 was immobilized on magnetic protein A Dynabeads (Thermo Fisher Scientific) and then pulldowns were carried out according to previously described protocols (38, 61). In addition to probing with α-Haus8, pulldown blots were probed using custom antibodies against *X. laevis* GST-importin-α and GST-importin-β generated by GenScript and purified from serum according to previously published protocols (38, 61).

### Tubulin labeling and polymerization of GMPCPP-stabilized MTs

Bovine brain tubulin was labeled following prior methods (62). Using Alexa568-NHS ester (Invitrogen; A20003) yielded 36 to 40% labeling efficiency. Single cycled GMPCPP-stabilized MTs were made as previously described (42, 63). Briefly, 12 μM unlabeled bovine tubulin supplemented with 1 μM Alexa568 tubulin and 1 μM biotin tubulin was polymerized in BRB80 buffer (80 mM Pipes, 1 mM EGTA, and 1 mM MgCl_2_) in the presence of 1 mM GMPCPP for 1 h at 37 °C. After 1 h, the MT seed mixture was centrifuged at 13,000*g* for 15 min. The supernatant was removed, and the pellet was resuspended in warm BRB80 buffer supplemented with 1 mM GMPCPP.

### Preparation of PEG-functionalized coverslips

About 22 mm × 22 mm cover glasses (Carl Zeiss; catalog no.: 474030-9020-000) were silanized and reacted with PEG as previously described (64), except that hydroxyl-PEG-3000-amine and biotin-PEG-3000-amine were used. Glass slides were passivated using poly(l-lysine)-PEG. Flow chambers for TIRF microscopy were prepared using parafilm and gentle heating to seal coverslips to the glass slides.

### Attachment of GMPCPP-stabilized MTs to PEG-functionalized coverslips

Flow chambers were incubated with 5% Pluronic F-127 in water (Invitrogen; catalog no.: P6866) for 5 min at room temperature and then washed with assay buffer (BRB80; 5 mM β-mercaptoethanol, 0.075% [w/v] methylcellulose, 1% [w/v] glucose, 0.02% [v/v] Brij-35 [Thermo Scientific; catalog no.: 20150]) supplemented with 50 μg/ml κ-casein. Flow chambers were then incubated with an assay buffer containing 50 μg/ml NeutrAvidin (Invitrogen; catalog no.: A2666) for 2 min on a metal block on ice and subsequently washed with BRB80. Next, flow chambers were incubated for 5 min at room temperature with Alexa568-labeled biotinylated GMPCPP-stabilized MTs diluted 1:2000 in BRB80. Unbound MTs were removed by additional BRB80 washes.

### Binding of proteins to GMPCPP-stabilized MTs

To assess the binding of augmin and Haus8 constructs to MTs, augmin and Haus8 were diluted to 100 nM in CSFxB with 100 mM KCl and added to the flow chamber containing GMPCPP-stabilized MT seeds, previously attached to the coverslip surface. This was incubated for 10 min at room temperature. Unbound proteins were washed away using BRB80. For experiments with importins, importin proteins were incubated with augmin proteins on ice for 10 min prior to entering the flow chamber. All samples were imaged immediately.

### TIRF microscopy and image analysis

TIRF microscopy was performed with a Nikon TiE microscope using a 100 × 1.49 numerical aperture objective. Andor Zyla cCMOS camera was used for acquisition, with a field of view 165.1 × 139.3 μM. Multicolor images were collected using the NIS-Elements software (Nikon). All adjustable parameters for imaging (exposure time, laser intensity, and TIRF angle) were kept the same within experiments. For *in vitro* experiments, the objective was warmed with an objective heater at 33 °C. Images belonging to the same experiment were contrast matched. Images used for quantification of MT binding were analyzed using ImageJ. To segment MTs, the tubulin signal was thresholded *via* the Otsu method. MTs were isolated from the mask by setting the minimum particle area as 0.5 μm^2^. Average fluorescent signal per pixel was recorded for each MT with and without additional proteins. To compare augmin fluorescence intensity across experiments, the intensity was normalized with respect to the tubulin signal.

### *Xenopus* egg extract reactions

Branching MT nucleation reactions were carried out in 5 μl volume flow cells with Alexa568-labeled fluorescent tubulin in the presence or the absence of Ran^Q69L^, as previously described (65). After 15 min, fixative (−20 °C methanol) was added and allowed to incubate for 1 min. This led to branched MTs adhered to the coverslip within each flow cell (hereafter called reactions). Fixative was washed out with a continuous flow of 50 μl blocking buffer (5% normal goat serum S1000 in low-salt CSFxB; Vector Labs) and then reactions were incubated in that buffer for 1 h at 4 °C. After this time, reactions were incubated overnight at 4 °C in blocking buffer with custom rabbit polyclonal α-Haus8. The following day, reactions were subjected to three rounds of washing with 50 μl low-salt CSFxB each followed by a 15 min incubation. Reactions were incubated for 1 h in blocking buffer containing goat α-rabbit IgG (H + L) secondary antibody Alexa Fluor 568 conjugate (Thermo Fisher Scientific). Again, reactions were washed with 50 μl low-salt CSFxB three times, and each wash was followed by a 15 min incubation. Finally, reactions were mounted with ProLong Diamond Antifade Mountant (Life Technologies), which was allowed to cure before imaging (typically >2 h). All steps and buffers were carried out at room temperature unless otherwise specified, and all incubations were performed in a humidity chamber.

## Data availability

All data are contained within the article.

## Supporting information

This article contains supporting information.

## Conflict of interest

The authors declare that they have no conflicts of interest with the contents of this article.
