# Supplementary material for: Augmin is a Ran-regulated spindle assembly factor
Source: J Biol Chem. 2023 Apr 21;299(6):104736. doi: 10.1016/j.jbc.2023.104736 (PMC10318467; doi:10.1016/j.jbc.2023.104736)
Supplement: Supporting Figure S1 [file mmc1.docx]

#

# Supplementary Information for

# RanGTP regulates the augmin complex

Kraus, Jodi^1†^; Travis, Sophie M^1†^; King, Matthew R^1,2^ Petry, Sabine^1*^

^1^Department of Molecular Biology; Princeton University; Princeton, NJ, 08544; USA

^2^Current address: Department of Biomedical Engineering; James McKelvey School of Engineering, Washington University in Saint Louis; Saint Louis, MO, 63130; USA

^†^Authors contributed equally to this paper

*Correspondence: spetry@princeton.edu

Preprint available on bioRxiv at: https://doi.org/10.1101/2022.12.23.521824

*******************************

*Xenopus laevis*  MSEA--G----VAPIEDGSQNSS---GGSSGDAALKKSKGGAKVVKSRYMQIGRSKVS-K 50

*Xenopus tropicalis*  MSEA--G----VAATEDGSQNSS---GGSSGDATLKKSKG-TKVVKSRYMQIGKSKVS-K 49

*Hymenochirus boettgeri*  MSEA--G----VTSLDDGAQNSS---GGSNEDAKFKKNK-GAKVIKSRYMQIAKSKVS-K 49

*Nanorana parkeri*  MAGEGEG----MAETGSGSQNSS---GASSGDTALKKKKAAPKMVKSRYMQYDKPKAAKK 53

*Rana temporaria*  MAGEGEG----MAELGSGSLNSS---GGSSGDVTVKKK-VPVKLVKSRYMQYK-PNIVKK 51

*Eleutherodactylus coqui*  MADSG------SNSLESGSQNSS---GGSSGDAAQKKV-KAPKVVKSRYMQYSKPKATKL 50

*Bufo bufo*  MAEPG------SDFLENGLQNSS---GGSSGDAALKKT-KGAKLVKSRYMQYKPKITK-- 48

*Bufo gargarizans*  MAEPG------SDFLENGLQNSS---GGSSGDGALKKP-KGAKLVKSRYMQYKPKITK-- 48

*Latimeria chalumnae*  MAGKGGGSSCGVSGSDSNILTRSQEDEAKSSGASLKKS-KGPKVVKSRYLDYDKNLSG-K 58

*Rhinatrema bivittatum*  MAEAG------VASLE------DGANGSGNNDPTVKKS-KGGKFVKARYLQYDKKKVG-K 46

*Geotrypetes seraphini*  MAEAG------AAPLE------DGANRSGSNDPPVRKS-KGGRFVKARYMQYDKKKLG-K 46

*Microcaecilia unicolor*  MAESG------VASLE------DGANRSGSNDPTVKKT-KGVRFVKARYMQYDKRKLG-K 46

*Columba livia*  MSA--------LANDS------GAT--VARGEASENKR-KGGRVVKSRYLDYDKKDAK-K 42

*Crocodylus porosus*  -----------MAARA------AGA--PAAAEGSGKRR-KGGRVVQSRYLQYERRVAD-K 39

*Platysternon megacephalum*  MAAAGS--PAGVPPGA------EGA--AEPDGSAGARP-KGGRVVKARYLQYDRKPVG-K 48

*Gekko japonicus*  ----------------------------MAENAPGAKV-RGGRIVPSRYLQYDRKTTG-K 30

*Vombatus ursinus*  MAEHR-SRPLGRSFSEAGPSV------SVNPKAKGRRV-PAGRIVESRYLQCEKKKVVKA 52

*Homo sapiens*  MADSS-GR--GAGKPATGPTN------SSSAKKKDKRV-QGGRVIESRYLQYEKKTTQKA 50

****

*Xenopus laevis* NSLANTTVCSGGKVPERGSGGTPTRRSLAPHKAKITAAVPLPALDGSIFTKEDLQSTLLD 110

*Xenopus tropicalis*  NSLANTTVCSGGKVPERGSGGTPTRRSVAPPKAKIAPGVPSVALDGSLFAKEDLQSTLLD 109

*Hymenochirus boettgeri*  NSLTNSTICTSGKVPEKGSGGTPTRRSIVPQKLRSIPGVPLTTLEGSLFVKDDLQSTLLD 109

*Nanorana parkeri*  PNVENSTLSSAGKAPDRGGNGTPTRRSVVPQRLKAPSAINASVADGNLFRKEDLQSTLLD 113

*Rana temporaria*  PNVENSIVSSAGKAQDRGGNGYPTRRSVLPQRFKAPSAVNASVAEVSLFSKAALQSTLLE 111

*Eleutherodactylus coqui*  KNNVANTTLSPGKGHERSRSGSL--KSVGLQRLKAALTPSN---ATDGSFKDDLQSTVLD 105

*Bufo bufo*  NNVVNTTPLQAGKSQEKSGSNTPTRRSVLPPRFKAPSATPN-VADGSSSFKEDLQSTLLD 107

*Bufo gargarizans*  NNVANTTPLQAGKSQEKSGSNTPTRRSVLPPRLKAPSATPN-VADGSSSFKEDLQSTLLD 107

*Latimeria chalumnae*  GPLVNAFSGSFSKPAEKG--RTPTQRSALPQKARPSACMQASALDYTPFGKDDLQSTLLE 116

*Rhinatrema bivittatum*  TNLINTI-SSGAKIPEKGRSGTPTRKSCILQKFKIPTRAESHVSDEALLRKDDLQSTLME 105

*Geotrypetes seraphini*  SNLVNTT-TSGAKVPEKGGSGTPTRKSCFLQKFKVPTGVMSNALDGAALSKDDLQSTLME 105

*Microcaecilia unicolor*  SNLVNSTAFSGAKIPEKAGSGTPTRKSCLLQKLKIPTAGVSNALDGAELSKNDLQSTLME 106

*Columba livia*  DNSASSFSTSVVKPSSGT-----KPRSALPQKSKKPADVASHSSSQSSFEKGDLQSTLLD 97

*Crocodylus porosus*  SILADSSTTSVGKGSEKE--GPVVGRSLLLQKCKAAAGIASGALNQSGLEKDDLQSTLLE 97

*Platysternon megacephalum*  STSANSSVVSGVKRSEKG--ETPTGRAGLFQKCKATTGTTPSTLNRTVLGKDDLQSTLLE 106

*Gekko japonicus*  ADIS----QSLMKEPERAASA--KRPPTQLQKQK----------NTSEMTFRVLHSTVLE 74

*Vombatus ursinus*  LASDTSINSSSGKQTESG------KKSTVSQKGS--TGVSAGSLATNQTEKGDLQSTLLE 104

*Homo sapiens*  PAGDGSQ--TRGKMSEGG------RKSSLLQKSK---------ADSSGVGKGDLQSTLLE 93

***********

*Xenopus laevis*  GHRIARPDLDLSVINDRTLQK--ITPRPVVTSEQKKPKRDTT---PVNLVPEDMVEMIES 165

*Xenopus tropicalis*  GHRIARPDLDLSVINDRTMQK--ITPRPAVTSEQKKPKRDTT---PVNIVPEDMVEMIES 164

*Hymenochirus boettgeri*  GHKIARPDLDLSVINDRTMQK--ITPRSLVTSDQKKHKKDST---PISIVPEDMVEMIES 164

*Nanorana parkeri*  GHKYVLPELDFSVINDKTLQK--LTPKSSSTSEQRKLKRETA---PVNNAPTDVIDMYES 168

*Rana temporaria*  GHKFIPPELDFSVINDR-------SPKCSSTSEQKKTKRKPA---PVNGVPIDMIDIYES 161

*Eleutherodactylus coqui*  GHKIARPELDFSVINDKTMQK--SSPKSLSTPAQRKSKRETT---SAVSIPEDTIEMLES 160

*Bufo bufo*  GHKIARPELDVSVINDKIMQK--LAQKSLSTSEHRKPKKEST---STTSIPEDMTDMYEL 162

*Bufo gargarizans*  GHKIARPELDVSLINDKTMQK--LTQKSLSTSEHRRPKKEST---STTSIPEDMTDMYEL 162

*Latimeria chalumnae*  GHRIALPDLDLSAINDKSLPKKGQTPETLFKVKAKNPKKELSKAILPPLPDQDLLEMIES 176

*Rhinatrema bivittatum*  GDKLERPDLDLSAVVNEKALHR-DIPKHLVKAEQRTRKKELI---MLPADPKGMIEILES 161

Geotrypetes seraphini GDKLVRPDLDLSAVINERTFHK-NAPKPLVKAEQRTPKKELL---LLPAVPEGMIEMLES 161

*Microcaecilia unicolor*  GDKLVRPDLDLSAVVNERTFHR-NTPKSLVKAEQRTPKMESL---PLPAVPEGMIGMLES 162

*Columba livia*  EDKISRPDLDISAISDKSVRKKTSASKSACKRNTRPQQKPK----EEGNDCDSLMEELES 153

*Crocodylus porosus*  GHKIVRPELDLSAINDKCLVRKTSTSKSVCEVDPRTRKKEQN---LKLSDPDDVIRMQES 154

*Platysternon megacephalum*  GHKIARPDLDLSAINDKSLLKKIPGSKSLFRAESRTRKKEQN---SKSSDPDDVIGMLES 163

*Gekko japonicus*  DHGNAQPDLDFSVISDKTRPAM-PLPKPNPAVKGT-SRKQLT---QMSPEAENLARLLES 129

*Vombatus ursinus*  GHRTAPPDLDLSAINDKSIKKRT--PQLTNKANTAQN---SKTPKKHNKVSTEDIKIMES 159

*Homo sapiens*  GHGTAPPDLDLSAINDKSIVKKT--PQLAKTISKKPESTSFSAPRKKSPDLSEAMEMMES 151

Supplemental Figure 1: Sequence alignment of the N-terminus of Haus8. Selected species include frogs (*Xenopus, Hymenochirus, Nanorana, Rana,* and *Eleutherodactylus*), toads (*Bufo*), other amphibians (*Latimeria, Rhinatrema, Geotrypetes,* and *Microcaecilia)*, as well as reptiles (*Columba, Crocodylus, Platysternon,* and *Gekko)* and mammals (*Vombatus* and *Homo sapiens*). Basic lysine and arginine residues are highlighted in blue. Note that, due to incomplete genome annotation, the first exon of *Gekko japonicus* is missing. NLS1 is indicated with yellow asterisks and NLS2 with green asterisks.
